# Safety and feasibility study of a novel robotic system in an in vivo porcine vascular model

**DOI:** 10.1186/s42155-024-00425-x

**Published:** 2024-01-27

**Authors:** Ornella Moschovaki-Zeiger, Nikolaos-Achilleas Arkoudis, Stavros Spiliopoulos

**Affiliations:** 2nd Department of Radiology, Interventional Radiology Unit, Medical School, National and Kapodistrian University of Athens, Attikon” University General Hospital, 1st Rimini St, Chaidari, 12461 Athens, GR Greece

**Keywords:** Robotics, Endovascular intervention, Visceral catheterization, Remote intervention, Robotic-assisted intervention

## Abstract

**Purpose:**

The goal of this preclinical study is to assess the functionality, technical feasibility, and safety of a new vascular robotic LIBERTY^R^ 3 System, in the microcatheterization of vascular targets using a range of guidewires and microcatheters.

**Material and methods:**

An anesthetized pig served as an arterial model for the robotic device (LIBERTY^R^3; Microbot Medical Ltd, Yoqneam, IL). The primary efficacy endpoint was the evaluation of its capability to selectively catheterize predetermined distal arterial branches in the liver, kidneys, and mesenteric arteries (technical success), under fluoroscopy guidance. The primary safety endpoint was the occurrence of angiographic acute catheterization-related complications (dissection, thrombosis, embolism, perforation). The catheterizations were conducted by two interventional radiologists that present different work experience in endovascular procedures (18 and 2 years respectively), using a variety of microcatheters and wires. Various procedural parameters such as functionality, practicality, ease of use, and time required for selective catheterization, were evaluated, and recorded.

**Results:**

All pre-determined arteries were successfully selectively catheterized (100% technical success), by both operators. No angiographic acute complications occurred. The microcatheters and wires were manipulated using the remote portable console in an effortless manner that maintained a high level of accuracy. Mean time for selective catheterization was 131 ± 82 s. The robot's conversion function to manual operation was successfully demonstrated.

**Conclusion:**

Robotic navigation and catheterization of selected target arteries were accomplished without observable vascular damage, suggesting that the LIBERTY^R^ 3 robotic system is a reliable and safe tool for robotic-assisted endovascular navigation. Further experimental studies are required to evaluate safety and efficacy prior to introduction into clinical practice.

## Background

The use of robotic surgical systems has increased substantially, from the first robotic system that was described and used by the National Aeronautics and Space Administration (NASA) [1], to the most prevailing and well-known telemanipulators, the Zeus and the da Vinci system, which have dominated the medical field for at least 10 years [2]. Recently, the advent of endovascular robotic systems, encouraged rapidly evolving international companies to develop a major element diversity to this globally increasing clinical reality. Experimental work suggests that robotic technology can be integrated with advanced localization, imaging techniques and AI which would improve performance even further.

With the use of robotic technology, endovascular procedures may now be performed remotely, eliminating radiation exposure for the operator, but also providing the possibility of a high degree of control while allowing to perform procedures from a comfortable posture, reducing the risk of occupational hazards. Studies have shown that interventionalists are particularly affected by increased rates of musculoskeletal distress and injuries [3], which could be prevented with the development and incorporation of consoles, that would enable operators to remain seated while carrying out procedures [4], without the need for the additional weight-bearing of the protective gear. Since the principal radiation protection measures and shielding used during endovascular interventions performed in the angiography suite are mostly operator-dependent [5, 6], the inclusion of a joystick-operated robotic device is a protective factor, as interventionalists will be able to operate from a safe distance in regard to the exposure of dispersed radiation. In comparison to merely skilled human performance, robotic systems have the advantage to assist navigation accuracy, dexterity, and speed [7] When it comes to delivering devices and manipulating catheters during robotic-assisted endovascular procedures, the early generation of devices required dedicated catheters (robotic catheters) and high-profile sheaths, which limit the practicality and increases the cost of consumables, especially in more complicated procedures [8, 9]. Newer generation vascular system technologies have undergone further technological development making possible to use off-the-shelf products, which makes them more appealing and accessible, laying the groundwork for the establishment of telemedicine and telementoring programs. The purpose of this pilot experimental study was to evaluate the technical efficacy and safety of the novel miniature, single-use (disposable), sterile, remote-controlled robotic system.

## Material and methods

### Robotic system

**Fig. 1:**
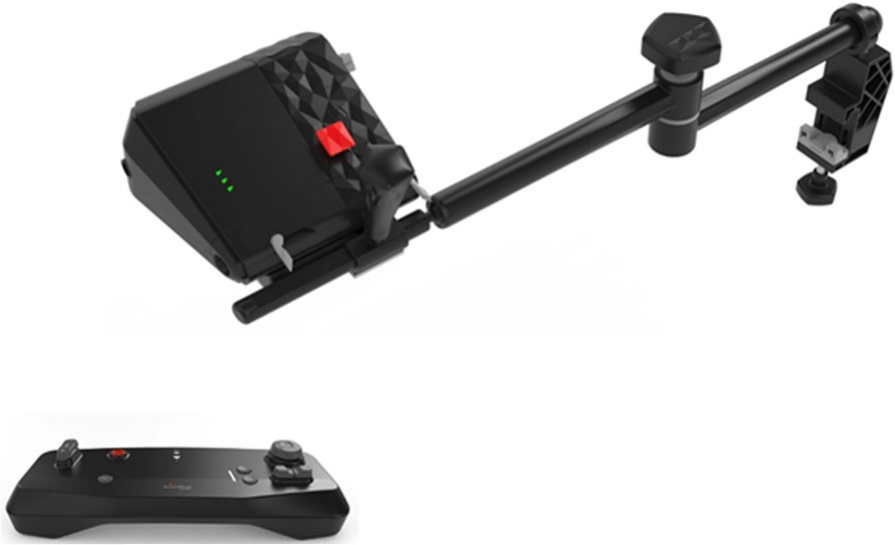
The liberty bedside robotic drive mounted on the mounting arm, and the hand-held remote-controller

**Fig. 2:**
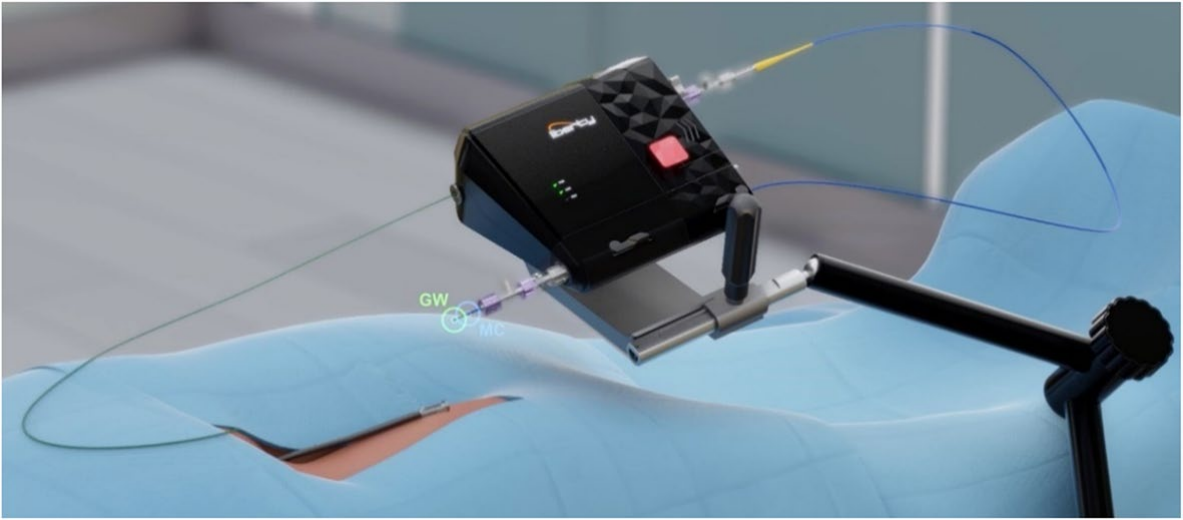
Schematic drawing illustrating the clinical set-up of the liberty system components, including the bedside robotic drive, being mounted on the mounting arm on the patient bed, and being operated remotely via the hand-held remote-controller (not shown). GW = Guidewire, MC = Microcatheter

The device that was tested was the Liberty^R^ 3 system (Microrobot Medical Ltd, Yoqneam, IL). The Liberty^R^ 3 is a non-commercially available, new endovascular robotic system, currently undergoing pre-clinical evaluation. Figures 1 and 2 display the primary components, which comprise of the bedside robotic drive (battery operated), the hand-held remote-controller unit, and the bedside-mounted articulated robotic arm. With the exception of the robotic arm which is composed of three foldable parts, with respective lengths of 25 cm, 25 cm and 20 cm, allowing the convenient and flexible usage, each of the components is designed for single-usage and is completely disposable. The weight of each component does not exceed 1.5 kgr (0.8 kgr for the robotic drive, 0.3 kgr for the remote control and 1.5 kgr for the robotic arm).

### Porcine model

In compliance with international laws for the protection of laboratory animals, the procedure was carried out at a certified animal laboratory. Veterinary professionals intubated and performed general anaesthesia to a single 60kg domestic pig, that was placed in supine position. At the completion of the experiment, all the contents were withdrawn, hemostasis was achieved with puncture site manual compression, and the animal was humanely euthanized.

### Endpoints and definitions

The study’s primary efficacy endpoint was technical success defined as the capability to catheterize predetermined distal arterial branches (*n* = 11) in the kidneys (upper, interpolar, lower pole branch of the right and left renal artery), the liver (3rd generation branch of the right and left hepatic artery), and mesenteric arteries (three 3rd generation branches of the superior mesenteric artery). The selective catheterizations were performed under fluoroscopy guidance, by manipulating a range of universal micro guide wires and catheters widely used in coil and liquid embolization, in both linear and rotational motion, and at varying speeds. The primary safety endpoints were the visual estimation of the occurrence of angiographic acute catheterization-related complications (dissection, thrombosis, embolism, perforation) and was compared to manual non robotic manipulations, and the evaluation of the immediate disconnection mechanism of the robot to allow manual operation.

Further parameters evaluated were: (a) characterization of the ease of use and operational performance of the Liberty 3 System by the 2 specialty physicians using prespecified scoring criteria (ease of remote control configuration, ease of guide wire and catheter manipulations) on a 1 to 5 scale (5 = optimal) to obtain a subjective assessment from the operator, and intraprocedural parameters while selectively catheterizing distal arterial branches within the liver, kidneys and mesenteric vessels, (b) evaluation of the sensitivity and stability of linear and rotational motion of the guide-wire, particularly the control at different speeds using the same 1 to 5 score (c) evaluation of the performance of the remote control, (d) evaluation of technical parameter including the mounting of the robot to the table, the loading sequence of micro catheter and guide wire into the robot and the mounting of the guide catheter holder bridging the vascular sheath and the robot, (e) time required for the microcatheterization of each predetermined vessel. Two interventional radiologists with 18 and 2 years of experience in endovascular procedures and with no prior experience with robotic systems, conducted the catheterizations.

### Procedure

The procedures were carried out in the animal lab using a C-arm by two radiologists (S.S., O.M.Z.) with 18 and 2 years of training and experience, respectively, in image-guided interventions, and naive to any robotic device including the Liberty^R^ 3.

Under sterile conditions and with ultrasound guidance, vascular access was obtained in the left common femoral artery with the use of an 8 Fr × 10 cm vascular sheath, that was sutured to the skin. Using a C2 Cobra hydrophilic catheter (Terumo, Japan), the right renal artery was manually catheterized. The proper catheter placement was verified with a control angiogram. The Liberty was loaded with a 2.4 Fr Pro-great microcatheter over a 0.016’ guidewire (Terumo, Japan) and the guiding catheter holder was used to attach the loaded Liberty to the C2 Cobra catheter, using the guiding catheter holder. With the use of the joystick, the robotic system was guided to the distal upper pole, interpolar, and lower pole branches of the right renal artery. Tasks involving intensive robotic manipulation of the microcatheter and micro guidewire at these sites were performed as part of the study. After the successful robotically guided catheterization, the microcatheter and wire were retracted to the proximal portion of the artery, and a diagnostic angiography was conducted. Both interventional radiologists executed the same process on the distal left renal artery branches.

In order to manually selectively catheterize the celiac trunk, a 0.035-inch guidewire and a 5 Fr C2 hydrophilic catheter was used. After manually delivering the selective catheter into the common hepatic artery, a 2.4 Fr Pro-great microcatheter with the 0.016’ GT guidewire was installed into the Liberty and attached to the C2 catheter. Robotic steering to the distal 3^rd^ generation right hepatic artery and subsequent relocation of the microcatheter system to distal 3^rd^ generation branches in the left liver lobe were both successfully accomplished. The guidewire and microcatheter were robotically rapidly retracted as part of the experiment, in the event of a code blue. The C2 catheter was then withdrawn in the aorta.

Using the same 5 Fr C2 hydrophilic catheter, manual selective catheterization of the proximal superior mesenteric artery was conducted, and a selective angiography verified the correct placement. To access the three distal 3^rd^ generation branches of the superior mesenteric artery, a 2.7 Fr ASAHI guidewire pre-loaded microcatheter was attached to the Liberty and coaxially placed into the guiding catheter, following successful navigation to the distal portions of the artery. After retracting the microcatheter and guidewire, a control angiography was carried out to verify that no acute angiographic complications had occurred during the procedure. The robot's capacity of rapid transition to manual operation was also tested. This was done by evaluating how rapidly and efficiently the instruments can be disconnected from their placement in the robotic system.

## Results

Both operators were able to effectively selectively catheterize all the pre-determined arteries, resulting in a 100% technical success rate, while no evidence of angiographically acute complications were indicated. Despite the lack of prior experience of both interventionalists with the robotic platform, performing superselective catheterization of the different vascular territories using the remote control demonstrated an intuitive demeanour with a steep learning curve. A high degree of precision was documented while the catheters and wires were handled with ease with the robotic control panel, with a minimum selective catheterization time of 49 s and a maximum of 213 s (mean selective catheterization time 131 ± 82 s). There was no statistically significant difference between the catheterization times recorded for each operator. An uninterrupted function was demonstrated through the observation that the functions of the robot may be controlled remotely from a range of distances within the operating room. The capability of the robot to rapidly convert from automatic to manual operation was effectively tested three times and the experiment was successfully terminated. Both operators reported optimal ease of use and sensitivity and stability scores, (median 5; range 4 to 5) No issues were noted regarding the predetermined technical parameters (performance of the remote control, mounting of the robot to the table, loading sequence of micro catheter and guide wire into the robot and the mounting of the guide catheter holder bridging the vascular sheath and the robot).

## Discussion

Using the Liberty^R^ 3 Robotic System, endovascular visceral super selective catheterizations, using standard endovascular micro catheter and micro guidewires, were conducted successfully in a porcine vascular model. The system offered a significant degree of practicality and high precision, as well as a fast response when remotely maneuvering and navigating the microcatheter system. Additionally, the console and joystick of the system, was assessed as simple to function and received very high satisfaction scores from the performing physicians. The safety of the robotic system was also demonstrated as no complications occurred. However, while using such a device, the absence of force feedback and haptic perception, which leads one to speculate that it may have the potential to lead to unintentional vascular injuries, if these procedures were performed in a larger study population. Nonetheless, a considerable reduction in risk may result from upgrades in systems for collision detection and the improvements of force sensor systems may also assist to mitigate these dangers [7]. According to this initial experience, the specific robotic system demonstrated a shallow learning curve for untrained operators, as demonstrated by the technical success rate and the short catheterization times by both operators. Moreover, the level of experience in endovascular procedures did not seem to influence the ability and time to catheterization, as both operators achieved similar results despite the vast difference in years of experience. One could speculate that similar results between the experienced and less experienced operator, could be noted as endovascular experience is challenged by the fact that experienced operators have developed manual automatizations in catheterization which are not easy to abolish, while younger operators, less familiar with manual manipulations, find it easier to adopt to remote control movements.

The incorporation of robotic systems in endovascular clinical practice could offer two significant advantages. The first is the optimization of complication-free technical success rates due to more finite and precise manoeuvres that can be performed even by operators with less years of endovascular experience As reported for previously investigated robotic systems, the Liberty^R^ 3 offers the possibility to accurately advance the micro catheter/guidewire in different speed levels but also perform a variety of precise angulations, two features that could increase catheterization success, while decreasing time to catheterization [10]. Nevertheless, comparative studies versus standard manual catheterization are required to prove such superiority. The second obvious advantage is the reduction of radiation-related occupational hazards for the operator and perhaps for the patients (as a result of reduced time to catheterization) [10–12]. As an additional advantage, the option to perform the interventions in sitting position throughout the procedure, would most probably lessen the impact that standing for lengthy periods of time with heavy equipment has on the body. Moreover, remotely controlled platforms are being developed so that operators may be located in large geographical distance from their patients, enabling for the delivery of procedures and skills to be carried out in facilities without an experienced endovascular professional [13].

Recently developed and marketed endovascular interventional robotic systems, that are widely recognized include the Corindus CorPath, the Hansen’s Magellan, and Robocath’ s R-One robot. The Corindus CorPath series (Corindus, Siemens Healthineers, Waltham, MA, USA), which was initially the sole commercially accessible robotic platform for coronary endovascular procedures, as developed by Beyar et al. [14], has pivoted its focus to cater exclusively to the domain of neurovascular interventions [15], following initial trials involving cerebral angiographies and carotid artery stenting [16]. A controllable dedicated robotic bending catheter was developed by the Hansen’s Magellan system (Hansen Medical, Mountain View, CA, USA) [17, 18], however it appears to have a high cost and provides no haptic feedback to the user [18]. The R-One robotic system by Robocath (Robocath, Rouen, France) is a distinct robotic platform with the capability to manipulate commercially available guidewires and stent and balloon catheters. However, it is limited as it cannot handle guiding catheters, while presently, it is exclusively employed in coronary interventions [19]. Among the robotic systems designed exclusively for cardiac interventions are the Niobe ES (Stereotaxis Inc., MO, USA), utilizing magnetic fields for navigation, and the Amigo (Catheter Precision, Inc., Mount Olive, NJ, USA), tailored for electrophysiological interventions such as cardiac ablation [19]. The main barriers for wide adoption of these endovascular robots are the size, weight and cumbersome set up of the robotic system, as well as the purchasing of capital equipment.

Notably, the Liberty^R^ 3 system offers three major advantages compared to other endovascular robotic systems. It has been designed as an aseptic single-use system that eliminates the risk of cross contamination and the need for sterilization. Additionally, the size and weight of the system enables easy transportation and provides ergonomic advantages that make it suitable for every standard angiography suite. Another advantage is its compatibility with standard off-the-shelf instruments and although this is a common feature with the Robocath R-One robot system, the latter is substantially larger and more expensive than the former [7]. Another essential feature of the robotic system is the capacity to transition to human control rapidly, thus increasing its safety profile. On the other hand, the incompatibility of the Liberty system with higher profile instruments is remains a disadvantage associated with this technology.

Establishing the efficiency, safety, financial advantages, and clinical results of robotic interventions is essential to overcome the obstacles associated as to introduce them in everyday clinical practice. Single-use concept and remarkably small size are the two unique features of the Liberty^R^ 3 robotic system that could overcome these obstacles.

The main limitation of this safety and feasibility experimental study is the small sample number of catheterized vessels, which as indicated, calls for additional research and data collection. Additionally, the study was conducted in non-pathological vessels, limiting the generalization of the findings to pathological vessels. Furthermore, the investigation of radiation exposure dosage and reduction in fluoroscopy time as the operators become more familiar with the system were not investigated and remain endpoints of future studies.

## Conclusion

In this initial experience, robotic navigation and catheterization of selected target arteries were rapidly accomplished without observable vascular damage, suggesting that the Liberty^R^ 3 is a reliable and safe system for robotic-assisted endovascular navigation. The system was reported as easy to use and highly ergonomic. Further studies are required to evaluate safety and efficacy (Table 1).

**Table 1:** Catheterization time

	Catheterization time (seconds)	
	Interventional Radiologist #1 (S.S.)	Interventional Radiologist #2 (O.M.Z.)
RK—upper pole	149	155
RK—interpolar	200	213
RK—lower pole	102	80
LK – upper pole	180	190
LK – mid pole	135	104
LK – lower pole	58	75
RHA—3^rd^ generation branches	78	130
LHA – 3^rd^ generation branches	160	198
SMA—branch #1	92	73
SMA—branch #2	121	167
SMA—branch #3	67	49

*RK* Right Kidney, *LK* Left Kidney, *RHA* Right Hepatic Artery, *LHA* Left Hepatic Artery, *SMA* Superior Mesenteric Artery

## Data Availability

All data are available upon reasonable request.
